# ZEB1‐AS1 mediates bone metastasis through targeting miR‐320b/BMPR1A axis in lung cancer

**DOI:** 10.1111/crj.13770

**Published:** 2024-05-23

**Authors:** Nianxi Tan, Junyi Tang, Guang Chen, Weilin Jiang, Zhiqin Liu

**Affiliations:** ^1^ Department of Cardiothoracic Vascular Surgery Zhuzhou Hospital Affiliated to Xiangya School of Medicine, Central South University Zhuzhou Hunan China; ^2^ Department of Orthopedics Zhuzhou Hospital Affiliated to Xiangya School of Medicine, Central South University Zhuzhou Hunan China

**Keywords:** bone metastasis, lung cancer, miR‐320b, ZEB1‐AS1

## Abstract

**Objective:**

This study aimed to explore the role and regulatory mechanism of lncRNA ZEB1‐AS1 in lung cancer.

**Methods:**

The expression of ZEB1‐AS1 and miR‐320b was determined by qRT‐PCR. Cell viability, proliferation migration, and invasion were assessed using the CCK‐8, colony‐forming, and Transwell assay. EMT markers were quantified using western blot. The growth of subcutaneous tumor growth and metastatic bone tumors was evaluated in mouse model of lung cancer. Additionally, metastatic bone tumors were examined using H&E staining.

**Results:**

ZEB1‐AS1 expression was upregulated, while miR‐320b levels were downregulated in lung cancer. Knockdown of ZEB1‐AS1 resulted in a significant suppression of cell viability, proliferation, migration, invasion, and EMT in A549 cells. Furthermore, we confirmed the targeting relationship between ZEB1‐AS1 and miR‐320b, as well as between miR‐320b and BMPR1A. Our findings suggested that ZEB1‐AS1 regulated cell viability, proliferation, migration, and invasion, as well as EMT, in lung cancer cells by targeting the miR‐320b/BMPR1A axis. Moreover, our in vivo experiments confirmed that ZEB1‐AS1 mediated bone metastasis through targeting miR‐320b/BMPR1A axis in mice with lung cancer.

**Conclusion:**

ZEB1‐AS1 mediated bone metastasis through targeting miR‐320b/BMPR1A axis in lung cancer.

## Graphical abstract

lncRNA ZEB1‐AS1 expression was upregulated in lung cancer, which mediated bone metastasis of lung cancer through targeting miR‐320b/BMPR1A axis.

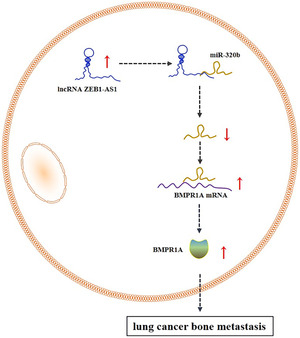

## 1 INTRODUCTION

Lung cancer is a prevalent malignant tumor worldwide, with bone metastasis being one of the most common sites for metastasis.^1^ Recent data show that up to 30%–40% of lung cancer patients may progress to bone metastasis, which can result in bone pain, fracture, spinal instability, and nerve compression.^2^ Radiation therapy is commonly used in the treatment for lung cancer patients with bone metastasis. However, these patients with bone metastasis have a relatively lower 1‐year survival rate of around 40%–50%.^3^ Therefore, it is of great significance to investigate the molecular mechanism underlying bone metastasis and identify potential therapeutic targets for bone metastasis in lung cancer.

Long noncoding RNAs (lncRNAs) are a class of RNA molecules longer than 200 nucleotides that interact with DNA, RNA, and proteins to modulate gene expression. In lung cancer, lncRNAs are involved in diverse biological functions, such as cell proliferation, invasion, and metastasis. As reported, the inhibition of lncRNA IGFBP4‐1 suppressed cell proliferation, induced apoptosis and regulated energy metabolism in lung cancer cells.^4^ Another study by Meng et al revealed that overexpression of lncRNA RMRP promoted cell proliferation, colony formation, and invasion by targeting miR‐206 in lung adenocarcinoma cell lines.^5^ Additionally, elevated expression of ZEB1‐AS1 was observed in non‐small cell lung cancer (NSCLC), and silencing ZEB1‐AS1 repressed cell viability and induced cell death.^6^ An in vitro study demonstrated that knocking down ZEB1‐AS1 inhibited cell growth, migration, invasion, and triggered apoptosis in NSCLC and the overexpression of ZEB1‐AS1 was correlated with poor prognosis in NSCLC patients.^7^ These studies illustrate that ZEB1‐AS1 aggravates the progression of lung cancer. However, the exact regulatory mechanism of ZEB1‐AS1 in bone metastasis in lung cancer still remains uncertain. Further investigation is needed to elucidate this mechanism.

MicroRNAs (miRNAs) play a crucial role in mediating physiological processes in lung cancers.^8^ For example, miR‐4317 suppressed cell proliferation and metastasis in NSCLC.^9^ Liang et al found that the upregulation of miR‐196b‐5p accelerated cell migration, proliferation, and cell cycle of lung cancer cell lines by targeting GATA6 and TSPAN12.^10^ Moreover, miR‐320b has been identified as a suppressor for lung cancer. Research by Ma et al proved the inhibiting impact of miR‐320b on lung cancer.^11^ Interestingly, bioinformatics analysis indicates that ZEB1‐AS1 acts as a competing endogenous RNA (ceRNA) for miR‐320b. However, the specific targeting relationship between ZEB1‐AS1 and miR‐320b has not been previously reported. Based on these findings, we speculated that ZEB1‐AS1 mediated bone metastasis in lung cancer by targeting miR‐320b. Recent evidence indicates that bone morphogenetic protein receptor, type 1A (BMPR1A) plays a role in osteogenesis and bone formation. It has been reported that ALK3/BMPR1A axis was targeted by USP15 in regulating bone morphogenetic protein signaling.^12^ Additionally, Ginsenosides Rg3 was found to enhance bone formation and decrease bone resorption by inhibiting the activation of BMP‐2/BMPR1A/Runx2 signaling in glucocorticoid‐induced osteoporosis.^13^ Nevertheless, the exact function of BMPR1A in bone metastasis in lung cancer still remains unclear. According to predictions from the TargetScan database, miR‐320b is predicted to target BMPR1A. Therefore, it is hypothesized that miR‐320b may mediate bone metastasis in lung cancer through targeting BMPR1A.

Thus, we speculated that ZEB1‐AS1 may regulate bone metastasis in lung cancer by targeting miR‐320b/BMPR1A axis. These findings might provide promising therapeutic target against bone metastasis in lung cancer.

## 2 MATERIAL AND METHODS

### 2.1 Patients and tissue samples

This study enrolled 20 cases of lung cancer patients with bone metastasis and 20 cases of lung patients without bone metastasis. Tissue samples were collected from the primary tumor, metastatic bone sites, and para‐cancerous tissues as controls. Including criteria were as follows: (1) Patients were newly diagnosed with primary lung cancer; (2) patients had not undergone chemotherapy or radiotherapy. Patients with other types of malignant tumors were excluded from the study.

### 2.2 Cell culture

BEAS‐2B cells and lung cancer cell lines, including A549, NCI‐H1299, Calu‐1, NCI‐H292, and HCC827 (ATCC, USA), were cultured in RPMI 1640 medium containing 10% FBS and 100 μg/ml penicillin–streptomycin (Thermo Fisher Scientific, USA). The cells were maintained at 37°C in a humidified atmosphere with 5% CO_2_.

### 2.3 Cell transfection and construction of plasmid

The overexpressing vector for ZEB1‐AS1 (pcDNA3.1‐ZEB1‐AS1), siRNA targeting ZEB1‐AS1 (sh‐ZEB1‐AS1), and siRNA targeting BMPR1A (sh‐BMPR1A), as well as negative control vectors, were synthesized by GenePharma (Shanghai, China). miRNAs including miR‐320b mimics, miR‐320b inhibitors, and negative controls (mimics NC, inhibitors NC) were bought from RiboBio (Guangzhou, China). For transfection, the cells were transfected with pcDNA3.1‐ZEB1‐AS1, sh‐ZEB1‐AS1, miR‐320b mimics, miR‐320b inhibitors, sh‐BMPR1A, or their corresponding negative controls using Lipo6000 reagent (Beyotime Biotechnology, China) for 48 h.

### 2.4 Transwell assay

To assess cell migration, the cell suspension (2.5 × 10^4^ cells/mL) was resuspended in serum‐free medium supplemented with 1% BSA (Sigma‐Aldrich) and placed in the upper chamber. In the lower chamber, 500 μL of DMEM and 10% FBS was added. After incubation for 24 h, the cells that migrated through the membrane were fixed and stained with 0.1% crystal violet. The number of migratory cells was then quantified using a microscope. For the determination of invasive abilities, a similar procedure was followed as for cell migration detection. However, in this case, 100 μL of diluted substrate gel (Sigma, USA) was added to the upper chamber before adding the cell suspension. The cells that invaded through the gel and migrated through the membrane were counted after the incubation period.

### 2.5 Tumor xenograft assay

Male BALB/c nude mice (SJA Laboratory Animal Company, China) were housed in a light‐controlled cage at 24°C. This study had been approved by the Institutional Animal Care Committee of Zhuzhou Hospital Affiliated to Xiangya School of Medicine. A549 cells transfected with adenovirus vector (sh‐ZEB1‐AS1 or sh‐NC) were subcutaneously implanted injected (1 × 10^6^/mice) into the right axilla and bilaterally into the tibial cavities. The mice were weighted every 5 days until 35 days post‐injection, and x‐rays were used to assess tibial metastasis and bone destruction. Subcutaneous tumors were collected upon sacrificing the mice, and the tumor weight was quantified.

### 2.6 H&E staining

Five‐micrometer sections of mouse tibia tissues were stained using hematoxylin–eosin (H&E) and fixed with neutral balata. The alterations in the tibia tissues were observed and analyzed using a microscope.

### 2.7 CCK‐8 assay

In brief, A549 cells were planted into 96‐well culture plates (2 × 10^3^ cells per well) and incubated overnight. Each well was then treated with 10 μL of CCK‐8 solution (Dojindo, Japan). After a 1‐h incubation, the absorbance of the solutions was measured at 490 nm.

### 2.8 Colony formation assay

Cell proliferation was assessed using colon formation assay as previously described.^14^ In brief, a total of 5 × 10^3^ A549 cells were seeded into each well of six‐well plates and cultured for 14 days. The cells were then fixed with glutaraldehyde and stained with 1% crystal violet for 15 min. Positive colony formation was photographed and counted using ImageJ software (NIH, USA).

### 2.9 qRT‐PCR

RNA extraction was performed using TRIzol reagent (Invitrogen, USA). The isolated total RNA was reversely transcribed to cDNA using a miRcute Plus miRNA First‐Strand cDNA Kit (Tiangen Biotech, China) for miRNA analysis. miRNA expression was analyzed on a 7900 HT Sequence Detection System using a MiRcute miRNA qPCR detection kit (SYBR Green) (Tiangen Biotech, China). For the detection of RNA, PrimeScript RT Master Mix (Takara Bio, Japan) was utilized to convert RNA into cDNA. Quantitation analysis of RNA expression was conducted on an ABI 7500 System (Applied Biosystems, USA) using TB Green® Premix Ex Taq™ II (Tli RNaseH Plus) (Takara, Japan). The primer sequences used in PCR are listed below:

BMPR1A F 5′‐CTTTACCACTGAAGAAGCCAGCT‐3′;
R 5′‐AGAGCTGAGTCCAGGAACCTGT‐3′;
miR‐320b F 5′‐GTCTCTTAGGCTTTCTCTTCCCA‐3′;
R 5′‐TTTTCCTTTTGCCCTCTCAACC‐3′;
ZEB1‐AS1 F 5′‐TCCCTGCTAAGCTTCCTTCAGTGT‐3′;
R 5′‐GACAGTGATCACTTTCATATCC‐3′;
GAPDH F 5′‐AAATCAAGTGGGGCGATGCT‐3′,
R 5′‐AGCCAAATTCGTTGTCATACTTCT‐3′;
U6 F 5′‐CGCTTCGGCAGCACATATAC‐3′;
R 5′‐ATTTGCGTGTCATCCTTGCG‐3′.

The relative quantitative analysis was carried out by the 2^−ΔΔCT^ method. U6 and GAPDH were served as the control.

### 2.10 Western blot

Protein extraction was performed, and proteins expression was evaluated using a BCA Protein Assay Kit (Beyotime, China). The samples were then separated by 8%–10% SDS‐PAGE and transferred onto PVDF membranes (Bio‐Rad, USA). After blocking with 5% defatted dry milk, the samples were incubated overnight at 4°C with primary antibodies against BMPR1A (ab264043, 1/500, Abcam, USA), E‐cadherin (ab269767, 1 μg/mL, Abcam, USA), N‐cadherin (ab76059, 1/1000, Abcam, USA), vimentin (ab8069, 1 μg/mL, Abcam, USA), fibronectin (ab268021, 1/1000, Abcam, USA), and snail (ab63568, 1/500, Abcam, USA). Subsequently, the samples were then incubated with goat anti‐rabbit IgG H&L (HRP) preadsorbed (ab97080, 1/10000, Abcam, USA) or rabbit anti‐mouse IgG H&L (HRP) (ab6728, 1/2000, Abcam, USA) at 37°C for 1 h. The brands were visualized using the SuperSignal West Pico Chemiluminescence system (Pierce, Inc. USA) and analyzed with ImageJ software (NIH).

### 2.11 Dual‐luciferase reporter assay

The potential binding sites between miR‐320b and ZEB1‐AS1 or BMPR1A were predicted using the DIANA database or starBase. To validate this interaction, wild‐type (WT) and mutant (MUT) fragments of ZEB1‐AS1 or BMPR1A were constructed, and their binding affinity with miR‐320b was examined. The WT and MUT fragments, encompassing the putative miR‐320b binding site regions, were cloned into the luciferase reporter vector pGL4.10 (Promega Corporation). A549 cells were treated with 100 ng of either WT or MUT plasmid, in combination with miR‐320b mimics or NC mimics, using Lipofectamine 3000 (Invitrogen). After 48 h of transfection, luciferase activity was measured using a dual‐luciferase reporter system (Promega), and the luciferase activity was normalized to the values of *Renilla* luciferase activity.

### 2.12 Statistical analysis

All data were expressed as mean ± SD and analyzed and drew with GraphPad Prism 7.0. Statistical comparison was performed using ANOVA or Student's *t*‐test. A *p*‐value <0.05 was considered to be statistically significant.

## 3 RESULTS

### 3.1 ZEB1‐AS1 is upregulated, and miR‐320b is downregulated in lung cancer

**FIGURE 1:**
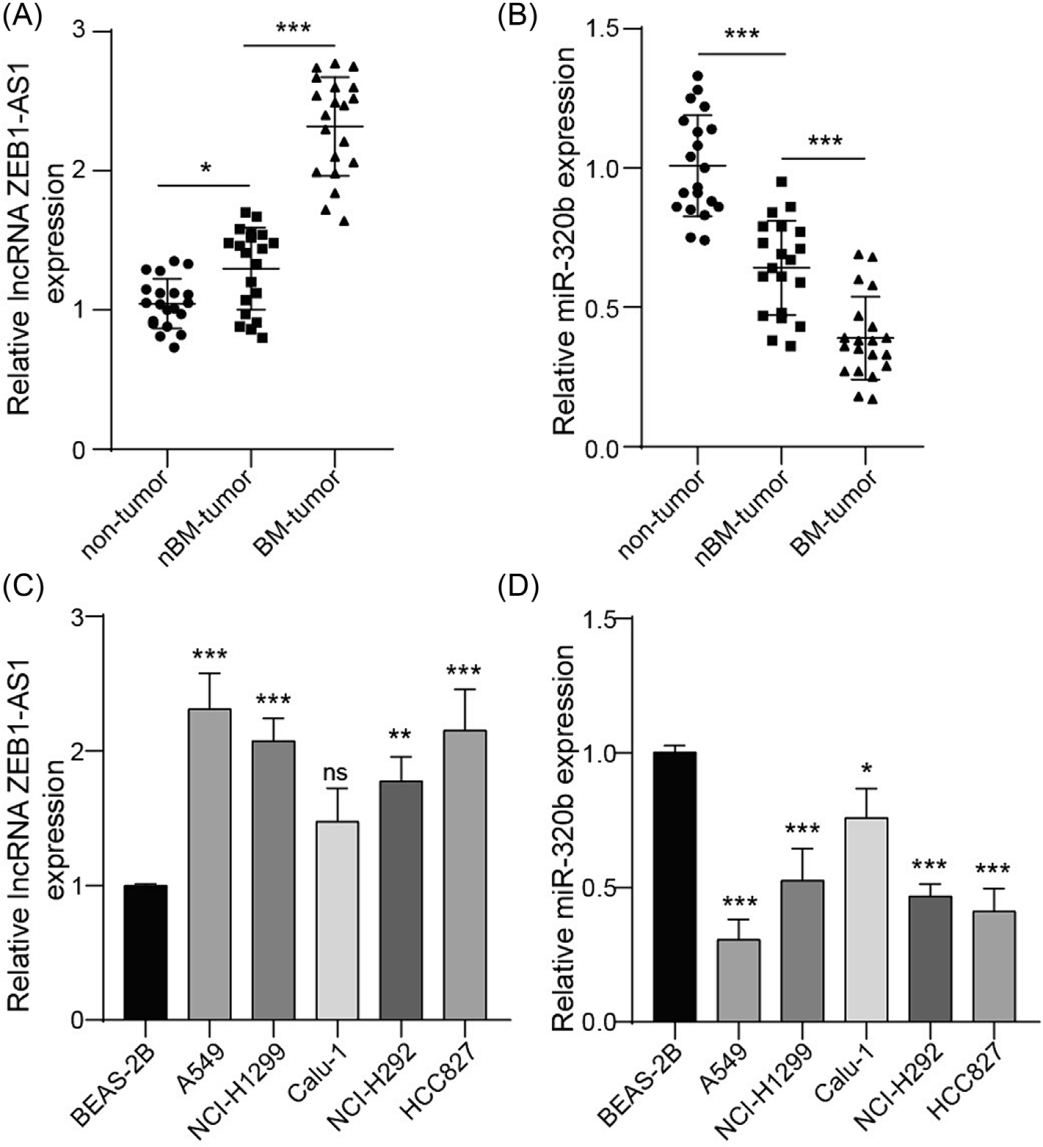
ZEB1‐AS1 was upregulated, and miR‐320b was downregulated in lung cancer. Para‐cancerous tissues (non‐tumor), primary tumor tissues (nBM‐tumor), and bone metastasis tissues (BM‐tumor) were collected (*n* = 20 for each group). (A) ZEB1‐AS1 and (B) miR‐320b levels were detected using qRT‐PCR. (C) ZEB1‐AS1 and (D) miR‐320b levels were measured using qRT‐PCR in human bronchial epithelial cell line BEAS‐2B and lung cancer cell line A549, NCI‐H1299, Calu‐1, NCI‐H292, and HCC827. *n* = 3, *p < 0.05, **p < 0.01, ***p < 0.001.

The expression levels of ZEB1‐AS1 and miR‐320b were detected in tissue samples obtained from patients with lung cancer. Compared with para‐cancerous tissues (nontumor), primary tumor tissues (nBM‐tumor) exhibited significantly higher levels of ZEB1‐AS1 expression, particularly in bone metastasis tissues (BM‐tumor). Conversely, decreased expression of miR‐320b was observed in the nBM‐tumor group, especially in BM‐tumor group (Figure 1A,B). To further investigate these findings, the expression of ZEB1‐AS1 and miR‐320b was evaluated in lung cancer cell lines. We observed upregulated levels of ZEB1‐AS1 and reduced levels of miR‐320b in lung cancer cell lines compared to the BEAS‐2B cell lines (Figure 1C,D). Notably, substantial alterations in ZEB1‐AS1 and miR‐320b expression were found in A549 cell line; thus, it was selected for the subsequent experiments.

### 3.2 Knockdown of ZEB1‐AS1 inhibits migratory and invasive capabilities of lung cancer cells

**FIGURE 2:**
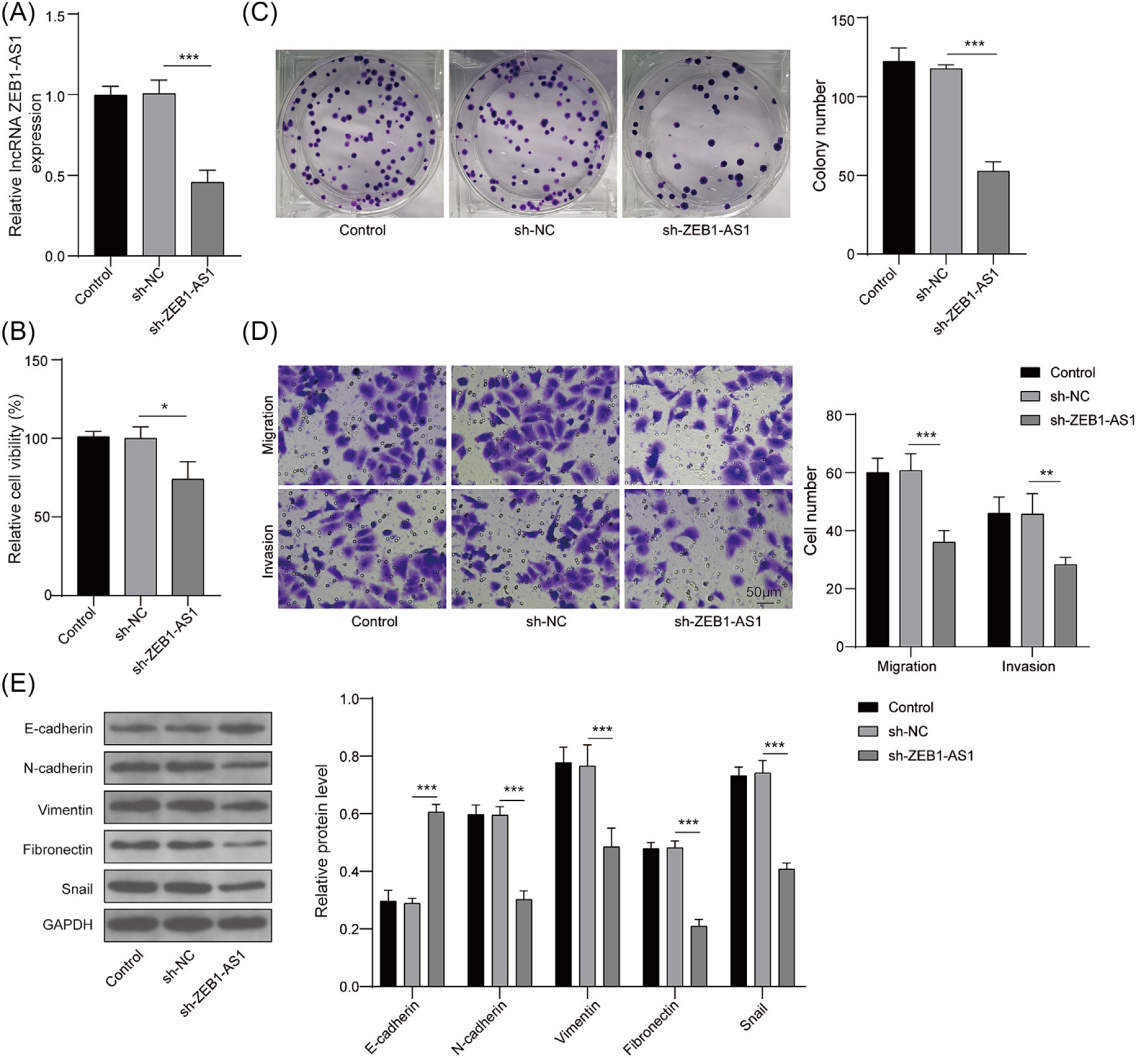
Knockdown of ZEB1‐AS1 inhibited migration and invasion of lung cancer cells. (A) A549 cells were transfected with sh‐ZEB1‐AS1 or sh‐NC. ZEB1‐AS1 expression was detected using qRT‐PCR. (B) Cell viability was evaluated using CCK‐8 assay. (C) Cell proliferation was assessed by colony‐forming assay. (D) Cell migration and invasion were determined using Transwell assay. (E) Protein levels of E‐cadherin, N‐cadherin, vimentin, fibronectin, and snail were measured by western blot. *n* = 3, *p < 0.05, **p < 0.01, ***p < 0.001.

The functional role of ZEB1‐AS1 was investigated in A549 cells. Initially, the transfection efficiency of sh‐ZEB1‐AS1 was confirmed in A549 cells. A screening of knockdown vectors for ZEB1‐AS1 was conducted (Figure S1A), and sh‐ZEB1‐AS1#1 was selected for subsequent experiments. As shown in Figure 2A, the expression of ZEB1‐AS1 was observed to be reduced by half in cells transfected with sh‐ZEB1‐AS1 compared to those transfected with sh‐NC. It was found that the cell viability and proliferation were remarkably repressed by the downregulation of ZEB1‐AS1 in A549 cells (Figure 2B,C). Furthermore, the knockdown of ZEB1‐AS1 exhibited significant suppressive effects on both cell migratory and invasive capabilities (Figure 2D). It was also observed that the depletion of ZEB1‐AS1 led to an increase in E‐cadherin protein levels in A549 cells while decreasing the protein levels of N‐cadherin, vimentin, fibronectin, and snail. This suggested that the silencing of ZEB1‐AS1 alleviated EMT process in lung cancer cells (Figure 2E). These findings collectively indicated that the down‐regulation of ZEB1‐AS1 effectively suppressed both the migratory and invasive capabilities of lung cancer cells.

### 3.3 ZEB1‐AS1 directly targets miR‐320b

**FIGURE 3:**
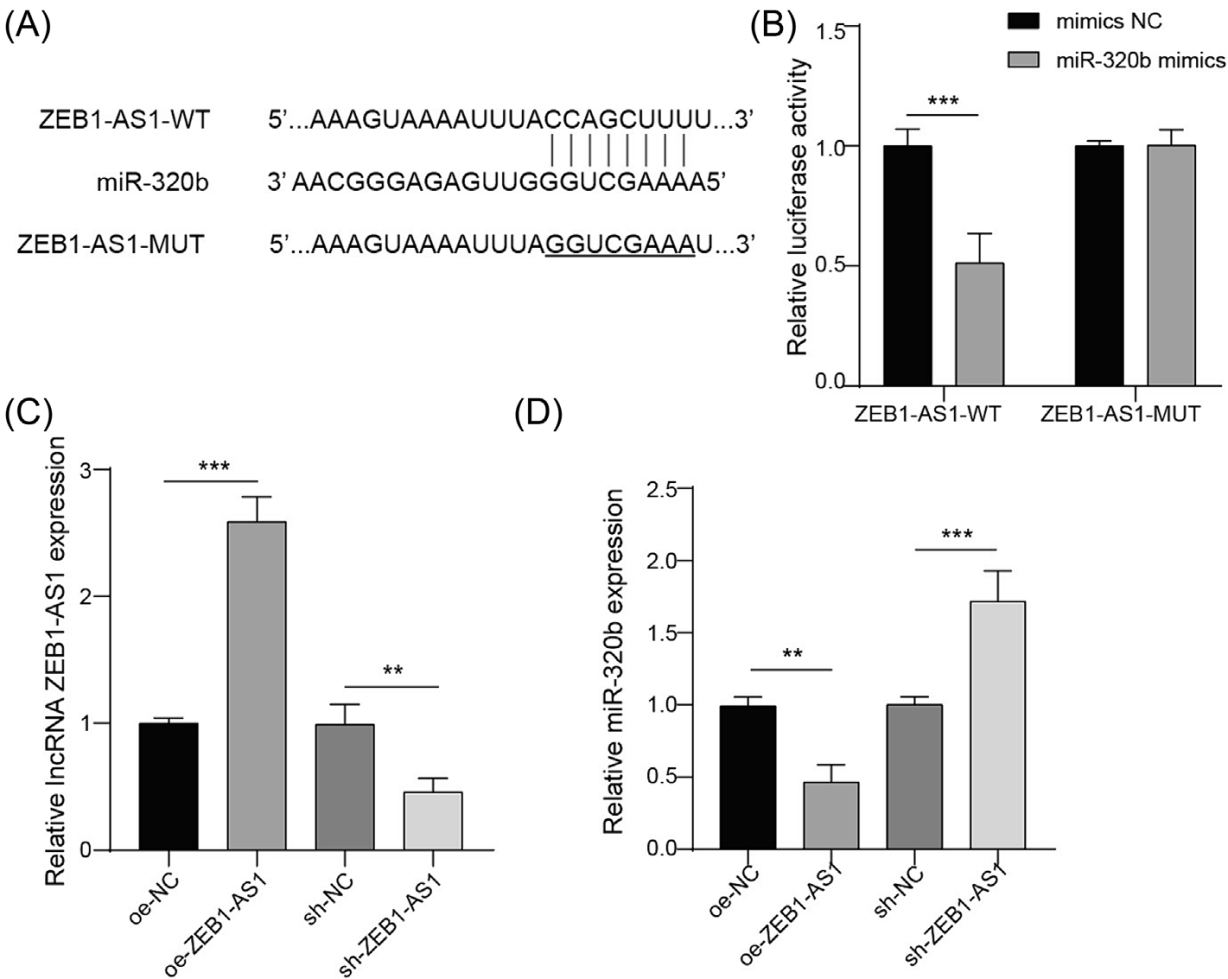
ZEB1‐AS1 directly targeted miR‐320b. (A) Biding site between ZEB1‐AS1 and miR‐320b was predicted by DIANA Tools database (http://diana.imis.athena‐innovation.gr/DianaTools/index.php). (B) Targeting relationship between ZEB1‐AS1 and miR‐320b was analyzed by dual‐luciferase reporter assay. (C) A549 cells were transfected with pcNDA3.1‐ZEB1‐AS1, sh‐ZEB1‐AS1, pcDNA3.1‐NC, or sh‐NC. Expression of ZEB1‐AS1 was calculated by qRT‐PCR. (D) Expression of miR‐320b was quantified by qRT‐PCR. *n* = 3, **p < 0.01, ***p < 0.001.

DIANA Tools database was utilized to predict the binding site between ZEB1‐AS1 and miR‐320b (Figure 3A). The results of dual‐luciferase reporter assay revealed that miR‐320b mimics induced a twofold reduction of fluorescence activity for ZEB1‐AS1‐WT, while no obvious regulation was observed for the fluorescence intensity of ZEB1‐AS1‐MUT (Figure 3B). To further investigate this interaction, the plasmid containing ZEB1‐AS1 or sh‐ZEB1‐AS1 was used to upregulate or downregulate ZEB1‐AS1 expression in A549 cells (Figure 3C). Subsequent qRT‐PCR analysis revealed that the overexpression of ZEB1‐AS1 notably decreased the expression of miR‐320b, whereas the downregulation of ZEB1‐AS1 increased its expression (Figure 3D). These findings indicated that ZEB1‐AS1 targeted miR‐320b and negatively regulated its expression.

### 3.4 miR‐320b directly targets BMPR1A

**FIGURE 4:**
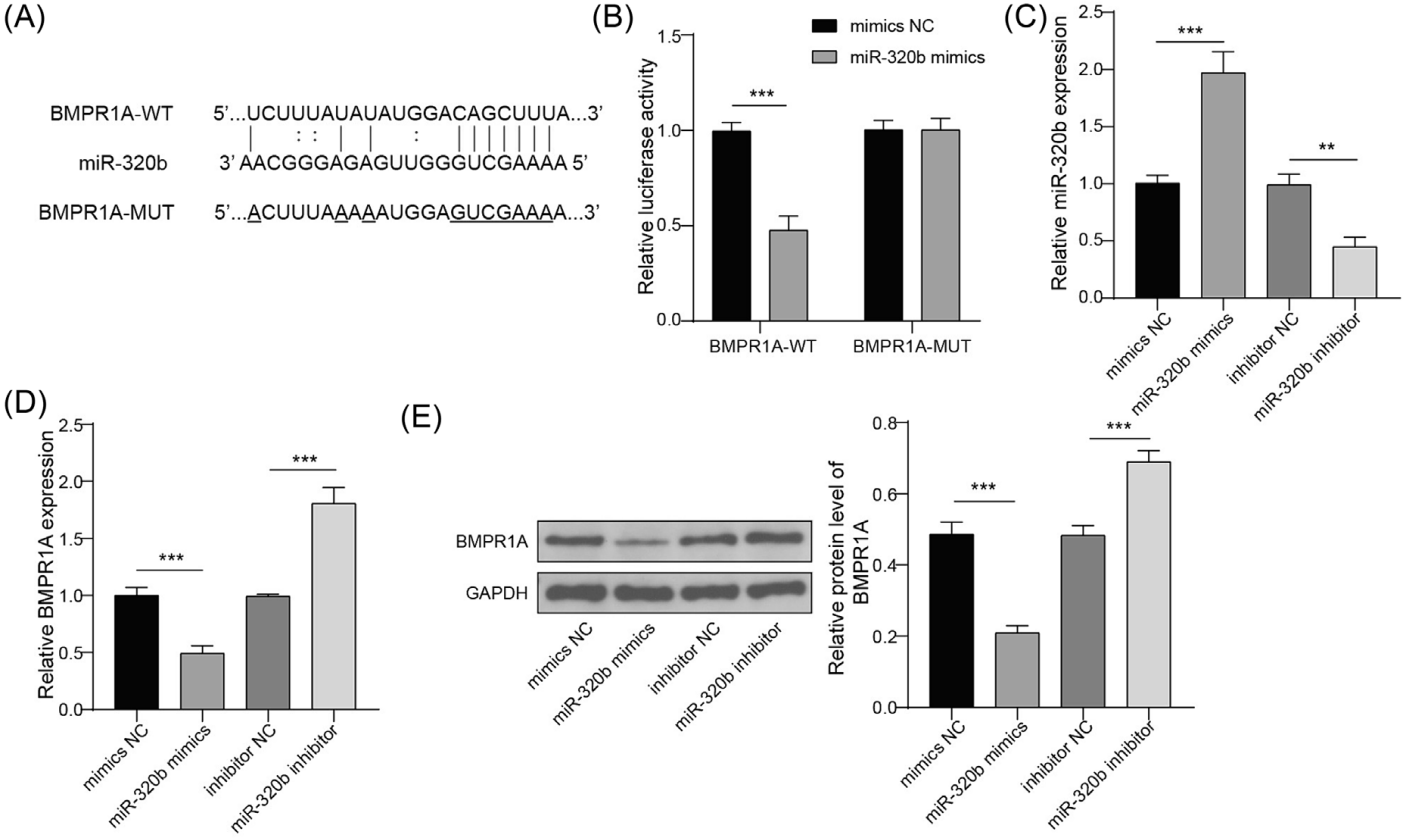
BMPR1A served as a target for miR‐320b. (A) Biding site between miR‐320b and BMPR1A was predicted by starBase (http://starbase.sysu.edu.cn/). (B) Targeting relationship between miR‐320b and BMPR1A was analyzed by dual‐luciferase reporter assay. (C) A549 cells were transfected with miR‐320b mimics, miR‐320b inhibitor, mimics NC, or inhibitor NC. Expression of miR‐320b was quantified by qRT‐PCR. (D) Expression of BMPR1A was quantified by qRT‐PCR. (E) Protein level of BMPR1A was analyzed using western blot. *n* = 3, **p < 0.01, ***p < 0.001.

According to the bioinformatics analysis of starBase, miR‐320b is predicted to bind to BMPR1A (Figure 4A). Subsequently, the interaction between miR‐320b and BMPR1A was verified by dual‐luciferase reporter assay (Figure 4B). We used miR‐320b mimics and miR‐320b inhibitor to regulate miR‐320b expression (Figure 4C), which showed obvious negative regulation for BMPR1A (Figure 4D). Consistent results were found in western blot analysis for BMPR1A content (Figure 4E). It could be concluded that miR‐320b bound to BMPR1A and negatively regulated its expression.

### 3.5 ZEB1‐AS1 regulates migration and invasion of lung cancer cells through miR‐320b/BMPR1A axis

**FIGURE 5:**
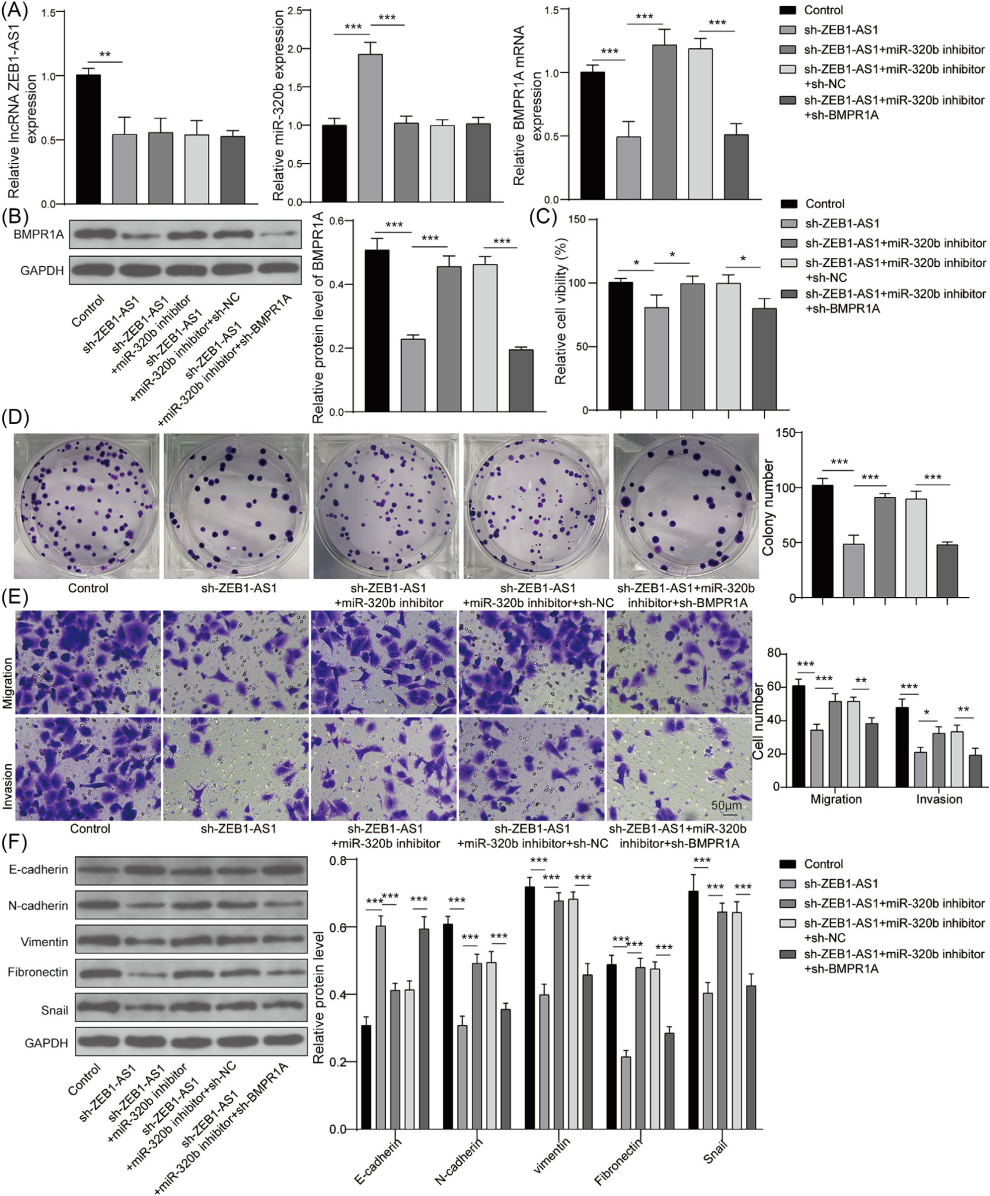
ZEB1‐AS1 mediated migration and invasion of lung cancer cells through miR‐320b/BMPR1A axis. A549 cells were transfected with sh‐ZEB1‐AS1, sh‐ZEB1‐AS1 + miR‐320b inhibitor, sh‐ZEB1‐AS1 + miR‐320b inhibitor + sh‐NC, or sh‐ZEB1‐AS1 + miR‐320b inhibitor + sh‐BMPR1A. (A) Expressions of ZEB1‐AS1, miR‐320b, and BMPR1A were calculated by qRT‐PCR. (B) BMPR1A protein was measured using western blot. (C) Cell viability was evaluated using CCK‐8 assay. (D) Cell proliferation was assessed by colony‐forming assay. (E) Cell migration and invasion were determined using transwell assay. (E) Protein levels of E‐cadherin, N‐cadherin, vimentin, fibronectin, and snail were detected by western blot. *n* = 3, *p < 0.05, **p < 0.01, ***p < 0.001.

The cellular pathway of ZEB1‐AS1 in the biological processes of lung cancer cells was studied. The knockdown vector of BMPR1A was screened (Figure S1B,C), and sh‐BMPR1A #2 was chosen for the subsequent experiments. The expression of ZEB1‐AS1 was remarkably reduced by sh‐ZEB1‐AS1, while no significant change in ZEB1‐AS1 levels was found in miR‐320b inhibitor or sh‐BMPR1A group. However, silencing ZEB1‐AS1 led to an increase in miR‐320b expression, which was reversed by co‐transfection with miR‐320b inhibitor but not affected by sh‐BMPR1A. Additionally, BMPR1A expression was repressed by ZEB1‐AS1 knockdown, and this suppression was counteracted by miR‐320b inhibitor but further decreased by co‐transfection with sh‐BMPR1A (Figure 5A). These results were further confirmed through western blot analysis (Figure 5B). It was found that both cell viability and proliferation were suppressed by ZEB1‐AS1 knockdown, but activated by the co‐transfection of miR‐320b inhibitor, which was further rescued by sh‐BMPR1A transfection (Figure 5C,D). Additionally, it revealed that sh‐ZEB1‐AS1 exerted a suppressive effect on the migration and invasion of A549 cells. In contrast, miR‐320b inhibitor intensified the migratory and invasive capabilities, and these effects were subsequently reversed by the co‐transfection of sh‐BMPR1A (Figure 5E). Moreover, a noticeable increase in E‐cadherin and a decrease in N‐cadherin, vimentin, fibronectin, and snail levels were observed in cells transfected with sh‐ZEB1‐AS1, which was rescued by miR‐320b knockdown. Meanwhile, sh‐BMPR1A inhibited the regulatory effect of miR‐320b knockdown on EMT markers, proving that ZEB1‐AS1 regulated EMT process of lung cancer cells through targeting miR‐320b/BMPR1A axis (Figure 5F). These findings demonstrated that ZEB1‐AS1 regulated mediated cellular functions in lung cancer cells by targeting miR‐320b/BMPR1A axis.

### 3.6 Knockdown of ZEB1‐AS1 inhibits bone metastasis in lung cancer mice

**FIGURE 6:**
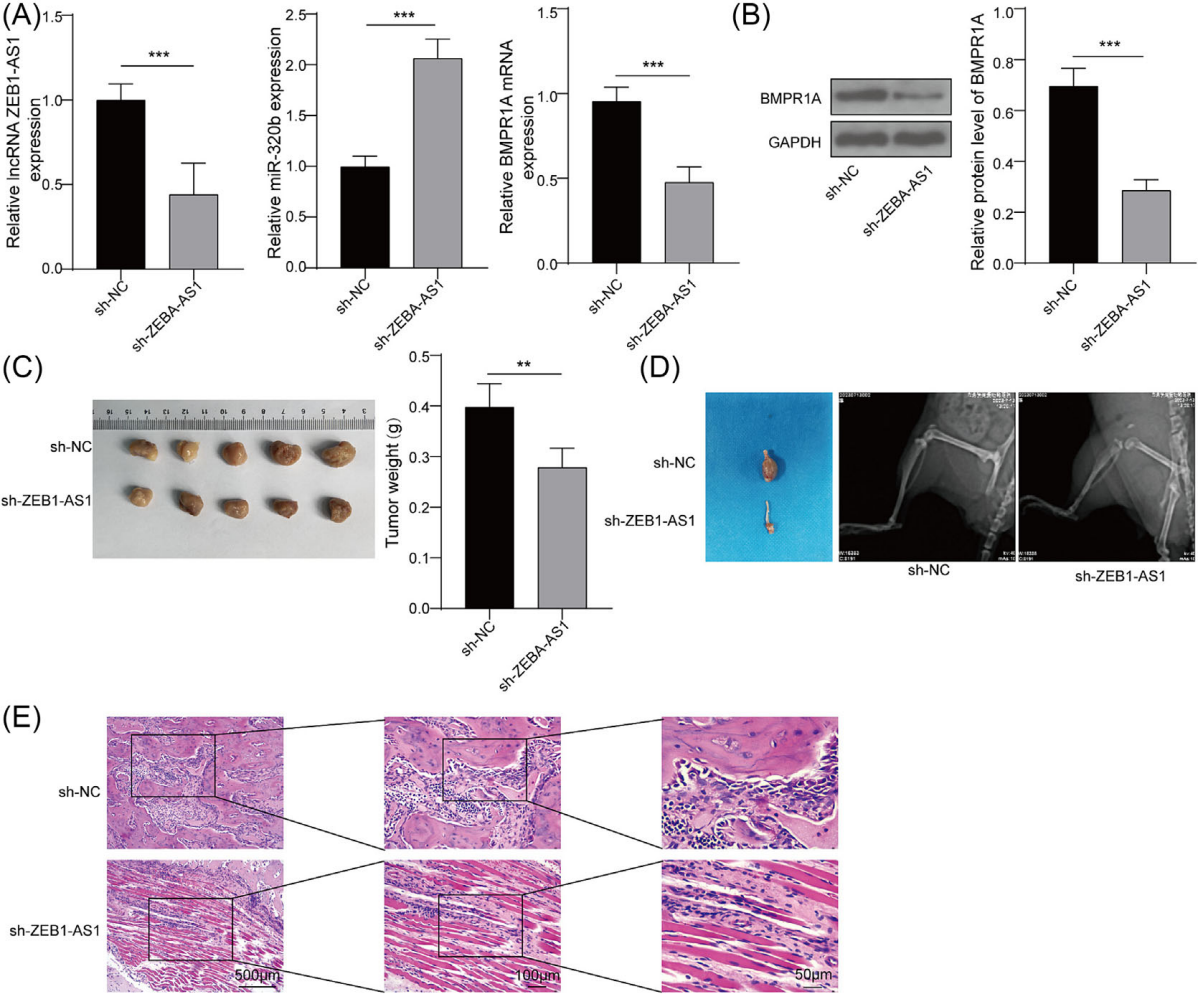
Knockdown of ZEB1‐AS1 inhibits bone metastasis in lung cancer mice. Lung cancer model was established in BALB/c nude mice. A549 cells stably expressing sh‐ZEB1‐AS1 or sh‐NC were implanted by subcutaneous injection into mice (*n* = 5 for each group). (A) Expressions of ZEB1‐AS1, miR‐320b, and BMPR1A in tumor tissues were calculated by qRT‐PCR. (B) Protein content of BMPR1A in tumor tissues was quantified by western blot. (C) The weights of subcutaneous tumors in mouse model were detected. (D) The tibial metastasis and bone destruction were assessed by x‐rays. (E) H&E staining images of tibia tissues were taken and analyzed. *n* = 5, **p < 0.01, ***p < 0.001.

Finally, the roles and potential mechanism of ZEB1‐AS1 on bone metastasis were also investigated using a mouse model of lung cancer. A549 cells stably expressing either sh‐ZEB1‐AS1 or sh‐NC were implanted via subcutaneous injection into mice. Figure 6A showed a decrease in ZEB1‐AS1 and BMPR1A levels, along with an approximately twofold increase in miR‐320b in the sh‐ZEB1‐AS1 group. Reduced ZEB1‐AS1 protein expression was further identified in the sh‐ZEB1‐AS1 group (Figure 6B). Subsequently, we measured the weights of primary tumors and metastatic bone tumors in mouse model. It was observed that both the weights of subcutaneous tumors and metastatic bone tumors were significantly reduced in sh‐ZEB1‐AS1 group (Figure 6C,D). H&E staining of tibial tissues revealed a decrease in the number of metastatic bone tumors following the inhibition of ZEB1‐AS1 compared to the control (Figure 6E). These findings demonstrated that the inhibition of ZEB1‐AS1 attenuated bone metastasis in mice with lung cancer.

## 4 DISCUSSION

Bone metastasis in lung cancer is influenced by factors such as the tumor's location, pathological type, microenvironment, and genetic attributes.^15^ Despite technological advancements and treatment methods for lung cancer, the clinical outcomes of patients with bone metastasis still remains unsatisfactory. Studies have indicated that elevated levels of ZEB1‐AS1 are associated with poor overall survival in NSCLC patients.^16^ Another study proved that increased ZEB1‐AS1 contributes to enhanced bone metastasis in hepatocellular carcinoma via miR‐302b/EGFR/PI3K/AKT signaling pathway.^17^ These findings led us to hypothesize that ZEB1‐AS1 might may serve as a key regulator of bone metastasis in lung cancer. Our study revealed that ZEB1‐AS1 mediated bone metastasis in lung cancer through targeting the miR‐320b/BMPR1A axis.

miRNAs play a significant role in the development of bone metastasis in lung cancer.^18^ For instance, miR‐33a was downregulated in lung cancer cells, acting as a suppressor of osteoclastogenesis by targeting parathyroid hormone‐related protein.^19^ Similarly, decreased levels of miR‐139‐5p were observed in lung adenocarcinoma patients with lytic bone metastasis. The in vivo study further identified that miR‐139‐5p promoted osteogenesis of mesenchymal stem cells by targeting Notch1.^20^ These studies suggest that miRNA might exert a suppressive effect on bone metastasis in lung cancer. However, Han et al demonstrated that miR‐106a accelerated cell migration and EMT process in lung adenocarcinoma through targeting TP53INP1, suggesting that miR‐106a exacerbated bone metastasis in lung cancer.^21^ In summary, miRNAs exhibit different functional roles in different tumors. Up to now, limited researches discuss the regulation of miR‐320b for bone metastasis in lung cancer. Our findings demonstrated that the suppression of miR‐320b accelerated the proliferation, migratory and invasive abilities, and EMT in A549 cells. Furthermore, ZEB1‐AS1 mediated bone metastasis by binding to miR‐320b in lung cancer. In line with these findings, a previous study demonstrated that lncRNA NR2F2‐AS1 accelerated proliferation and invasive capability and reduced cell death of lung cancer cell lines by targeting miR‐320b.^22^

BMPR1A is considered to be a promoter of tumor metastasis in various cancer types. A previous study revealed that LAPTM5 aggravated lung metastasis through mediating the lysosomal degradation of BMPR1A.^23^ Another study indicated that the silencing of BMPR1A suppressed osteoclastogenesis in breast cancer cells by inhibiting RANKL via p38 pathway.^24^ Similarly, it was reported that the inhibition of BMPR1A alleviated the formation and metastasis of breast cancer.^25^ Nevertheless, the specific functional role of BMPR1A has not been fully elucidated. Our study demonstrated that the deletion of BMPR1A suppressed viability, migratory and invasive abilities, and EMT process of lung cancer cell lines, indicating the oncogenic activity of BMPR1A in bone metastasis. Recent studies have highlighted the regulatory role of miRNAs on BMPR1A in tumor progression. For instance, miR‐885‐3p was found to regulate tumor angiogenesis by mediating BMPR1A,^26^ while Guo et al discovered that miR‐656 inhibited cell viability, neurosphere formation, and migration in glioma by downregulating BMPR1A.^27^ Consistent with these findings, our study suggested that ZEB1‐AS1 regulated miR‐320b/BMPR1A axis‐mediated bone metastasis in lung cancer cells.

In summary, our findings demonstrated that ZEB1‐AS1 mediated bone metastasis by targeting miR‐320b/BMPR1A axis in lung cancer. Our work might represent a promising approach for the prevention and treatment of bone metastasis in lung cancer patients.

## AUTHOR CONTRIBUTIONS

*Conceptualization*: Nianxi Tan and Zhiqin Liu. *Formal analysis*: Junyi Tang and Guang Chen. *Funding acquisition*: Weilin Jiang. *Investigation*: Guang Chen and Weilin Jiang. *Writing—original draft*: Nianxi Tan and Zhiqin Liu. *Writing—review and editing*: Nianxi Tan and Zhiqin Liu.

## CONFLICT OF INTEREST STATEMENT

The authors declare that they have no conflict of interest.

## ETHICS STATEMENT

This study has obtained the approval from the Medical Ethics Committee of Zhuzhou Hospital Affiliated to Xiangya School of Medicine. All participants signed the written informed consent.

## Supporting information


**Figure S1.** (A) A549 cells were transfected with sh‐NC, sh‐ZEB1‐AS1#1, sh‐ZEB1‐AS1#2 or sh‐ZEB1‐AS1#3. ZEB1‐AS1 expression was detected using qRT‐PCR. A549 cells were transfected with sh‐NC, sh‐BMPR1A#1, sh‐BMPR1A#2 or sh‐BMPR1A#3. (B) BMPR1A mRNA expression was detected by qRT‐PCR. (C) Protein level of BMPR1A was measured by western blot. n = 3, *p < 0.05, **p < 0.01, ***p < 0.001.

## Data Availability

The data that support the findings of this study are available from the corresponding author upon reasonable request.
