# Supplementary figures and images for: ZEB1‐AS1 mediates bone metastasis through targeting miR‐320b/BMPR1A axis in lung cancer
Source: Clin Respir J. 2024 May 23;18(5):e13770. doi: 10.1111/crj.13770 (PMC11116843; doi:10.1111/crj.13770)

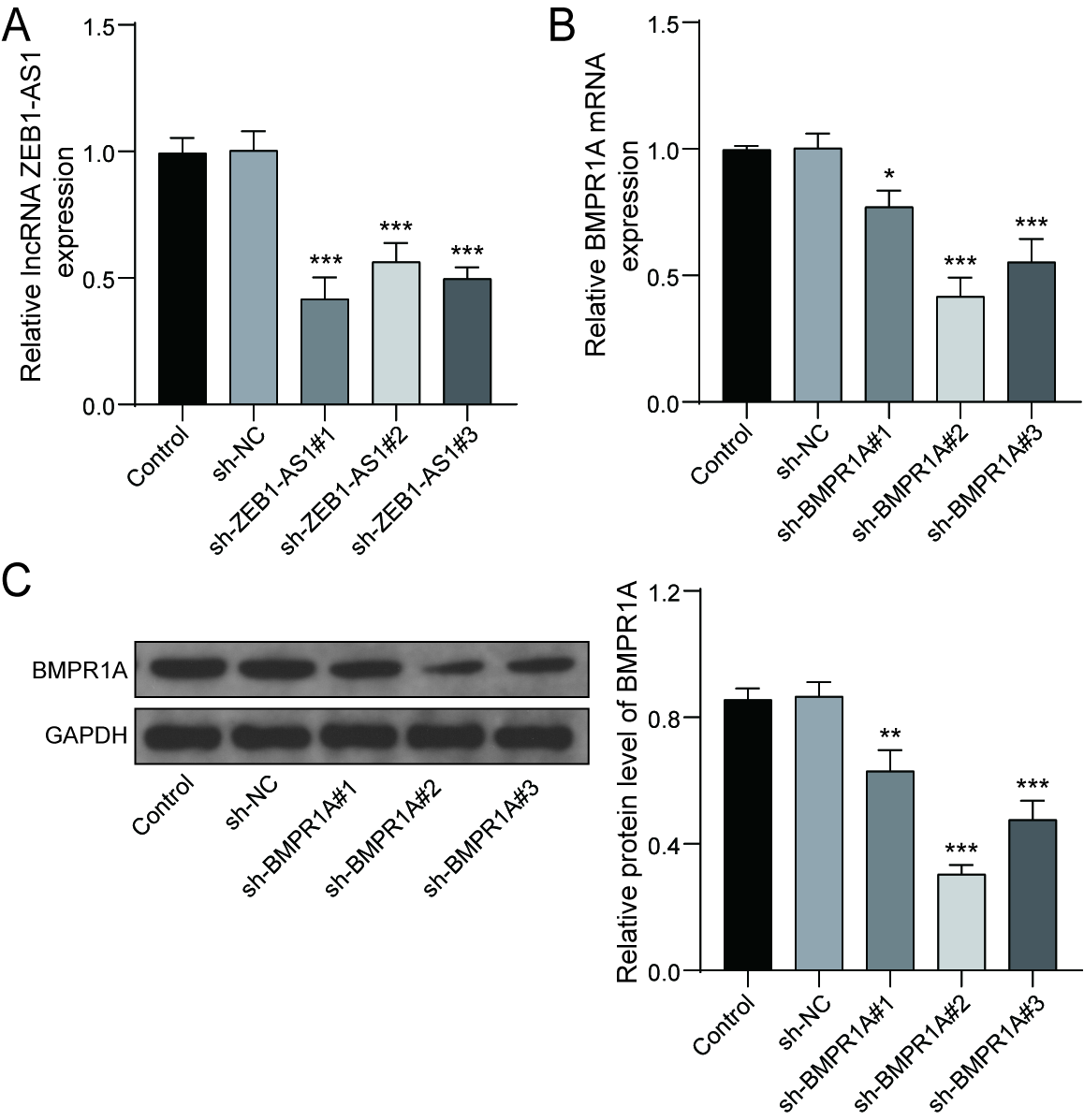

Supplement: Supplementary file 1 — Figure S1. (A) A549 cells were transfected with sh‐NC, sh‐ZEB1‐AS1#1, sh‐ZEB1‐AS1#2 or sh‐ZEB1‐AS1#3. ZEB1‐AS1 expression was detected using qRT‐PCR. A549 cells were transfected with sh‐NC, sh‐BMPR1A#1, sh‐BMPR1A#2 or sh‐BMPR1A#3. (B) BMPR1A mRNA expression was detected by qRT‐PCR. (C) Protein level of BMPR1A was measured by western blot. n = 3, *p < 0.05, **p < 0.01, ***p < 0.001. [file CRJ-18-e13770-s001.tif]
